# Approaches to minimising the epidemiological impact of sources of systematic and random variation that may affect biochemistry assay data in UK Biobank

**DOI:** 10.12688/wellcomeopenres.16171.2

**Published:** 2021-01-04

**Authors:** Naomi E. Allen, Matthew Arnold, Sarah Parish, Michael Hill, Simon Sheard, Howard Callen, Daniel Fry, Stewart Moffat, Mark Gordon, Samantha Welsh, Paul Elliott, Rory Collins

**Affiliations:** 1 Clinical Trial Service Unit and Epidemiological Studies Unit, Nuffield Department of Population Health, University of Oxford, OXFORD, Oxon, OX3 7LF, UK; 2 UK Biobank, Stockport, Cheshire, SK3 0SA, UK; 3 MRC Population Health Research Unit, Nuffield Department of Population Health, University of Oxford, OXFORD, UK; 4 MRC Centre for Environment and Health, Imperial College London, London, UK

**Keywords:** biochemistry, biomarkers, UK Biobank, cohort study, epidemiology

## Abstract

**Background**: UK Biobank is a large prospective study that recruited 500,000 participants aged 40 to 69 years, between 2006-2010.The study has collected (and continues to collect) extensive phenotypic and genomic data about its participants. In order to enhance further the value of the UK Biobank resource, a wide range of biochemistry markers were measured in all participants with an available biological sample. Here, we describe the approaches UK Biobank has taken to minimise error related to sample collection, processing, retrieval and assay measurement.

**Methods**: During routine quality control checks, the laboratory team observed that some assay results were lower than expected for samples acquired during certain time periods. Analyses were undertaken to identify and correct for the unexpected dilution identified during sample processing, and for expected error caused by laboratory drift of assay results.

**Results**: The vast majority (92%) of biochemistry serum assay results were assessed to be not materially affected by dilution, with an estimated difference in concentration of less than 1% (i.e. either lower or higher) than that expected if the sample were unaffected; 8.3% were estimated to be diluted by up to 10%; very few samples appeared to be diluted more than this. Biomarkers measured in urine (creatinine, microalbumin, sodium, potassium) and red blood cells (HbA1c) were not affected. In order to correct for laboratory variation over the assay period, all assay results were adjusted for date of assay, with the exception of those that had a high biological coefficient of variation or evident seasonal variability: vitamin D, lipoprotein (a), gamma glutamyltransferase, C-reactive protein and rheumatoid factor.

**Conclusions**: Rigorous approaches related to sample collection, processing, retrieval, assay measurement and data analysis have been taken to mitigate the impact of both systematic and random variation in epidemiological analyses that use the biochemistry assay data in UK Biobank.

## Introduction

UK Biobank is a population-based prospective study designed to allow the reliable assessment of a wide range of different types of exposure (including lifestyle, environment and genes) to multiple diseases, including those that cause much morbidity and disability but have not previously been extensively investigated ^1^. Recruitment into UK Biobank started in 2007 (following a successful pilot in 2006), and enrolment of 500,000 men and women aged 40 to 69 was achieved by mid-2010. UK Biobank’s scientific protocol and procedures have been approved by the North West NHS research ethics committee. The resource is also an approved Research Tissue Bank and is registered with the Human Tissue Authority, which means that researchers who wish to use it do not need to seek separate ethics approval (unless re-contact with participants is required).

The assessment visit included the collection of a vast amount of self-reported data via a touchscreen questionnaire and nurse interview, a wide range of physical measures (e.g. blood pressure, anthropometry, spirometry) and biological samples (blood, urine and saliva). Such data depth and breadth was made possible by implementing purposefully-designed, high-throughput processes. This included carefully piloted sample collection and processing protocols designed to collect and store participant samples for maximum scientific return over the long-term, and automation of sample aliquoting and storage to provide a consistent and fully auditable process.

Since recruitment ended in 2010, extensive data continue to be collected from participants. Subsets of the cohort are invited to have a repeat assessment every few years (the first of which was performed during 2012–13 on 20,000 participants and the second is ongoing during 2016–22 on 100,000 participants who are being imaged) to allow for correction of regression dilution bias caused by measurement error or intra-individual changes in exposures and biomarkers ^2^. A series of web-based questionnaires are routinely sent to all participants with an email address (n=330,000) to obtain further information about particular exposures (e.g. diet and food choices, occupation) and health-related conditions (e.g. cognition, mental health, pain). Other sub-studies designed to capture objective physical measures include physical activity monitoring (n=100,000 ^3^), ongoing assessments of multi-modal imaging (target of 100,000 ^4^); and cardiac monitoring (target of >30,000). Extensive assessment of exposures is combined with comprehensive follow-up and characterisation of many different health outcomes, achieved through linkage with electronic health records (including death and cancer registries, primary and secondary care data), as described in detail elsewhere ^5^.

One major way in which UK Biobank continues to enhance the utility of the resource is by converting the information contained in the biological samples, which are limited and depletable, into data that can be readily used by researchers worldwide from both academia and industry ^6^. Examples of cohort-wide assays that have been performed, or are ongoing, include genome-wide genotyping with subsequent imputation to over 90 million variants ^7^, exome ^8^ and whole genome sequencing, telomere length, and NMR-metabolomics. The aim is to make UK Biobank not only one of the largest prospective studies in the world but also one of the most detailed, with data on lifestyle, genetics, biomarkers and imaging.

In order to enhance the value of the UK Biobank resource, we sought to measure a wide range of biochemical markers in samples collected at baseline from all 500,000 participants and among the 20,000 participants who attended a repeat assessment visit 4–5 years later (2012–2013). The assays were selected by the UK Biobank Enhancements Working Group, with additional input from external experts as required. In total, 34 biomarkers were chosen based on their scientific relevance for studying a wide range of diseases, and included established risk factors for disease (e.g., lipids for vascular disease, sex hormones for cancer), diagnostic measures (e.g., HbA1c for diabetes and rheumatoid factor for arthritis) or markers of phenotypes that were not otherwise well assessed (e.g., renal and liver function). Measurement of all 34 assays was phased; the urine samples were assayed between Aug 2014 and Feb 2016, red blood cells (HbA1c) between Oct 2014 and March 2016, and the serum samples between Nov 2015 and Oct 2017. Investigation of the pre-analytical and analytical sources of error was examined and the data released in May 2019.

This article aims to describe the epidemiological considerations involved in collecting, storing, selecting, assaying and analysing a range of biochemistry markers of interest for research into common conditions at scale, the issues identified during this process, and to make recommendations on how best to utilise these data for research purposes.

## Methods

### Approaches used to reduce variation during sample collection and processing

During recruitment of 500,000 participants into UK Biobank, 2006–2010, a series of biological samples comprising blood (about 45 ml), urine (about 9 ml) and, for the last 85,000 participants, saliva were collected by a phlebotomist or a nurse. Blood and urine samples were not available for 0.3% and 1.8% of participants, respectively, because either they declined or it was not possible to collect for other reasons.

The samples were collected at various times throughout the day, depending on the time that participants attended the assessment centre. They were collected in different vessels so that a variety of preservatives, anti-coagulants and clot accelerators could be used to allow the widest possible range of assays that could plausibly be envisaged for the future, with detailed input from UK Biobank’s extensive academic collaborative network. The collection vessels (vacutainers and collection pots) were then processed on a variety of automation systems to create, for some sample types, multiple aliquots for long-term storage. Sample aliquots (comprising 850 µl sample within 1.4ml tubes) from each participant are stored in a fully automated -80°C working archive and in a manual, liquid nitrogen back-up archive located at a separate site ( Table 1). Any additional samples (such as aliquots of extracted DNA) are stored in manual freezers. This multi-storage approach provides protection from loss due to breakdown at a single site, minimises degradation caused by freeze-thawing, and enables measurement of analytes that are particularly sensitive to temperature over the long-term. Extracted DNA samples are stored in manual freezers.

**Table 1.** Sample collection and maximum number of aliquots created for each sample type.

Sample collection tube	Fractions	Number of aliquots (1.4 ml)	
		-80°C	-196°C
EDTA x2	Plasma	6	2
	Buffy coat	1	1
	Red cells	-	2
Lithium heparin (PST)	Plasma	3	1
Silica clot accelerator (SST)	Serum	3	1
Acid citrate dextrose	DMSO blood	-	2
EDTA	Haematology	-	-
Urine	Urine	4	2
Tempus tube (RNA)	Whole blood	6	-
Saliva	Mixed saliva	2	-
**Total**		**25**	**11**

EDTA: Ethylenediaminetetraacetic acid; PST, plasma separator tube; SST, serum separator tube.

The sample handling procedures ^9^ were the result of extensive consultation and piloting to ensure that they were likely to be fit for purpose and feasible at scale ^10^. For example, the pilot studies showed that, while compliance with providing a fasting sample was high, there was little difference in the time since last food recorded at the assessment centre. Moreover, a small number of participants who were advised not to fast (e.g., those with diabetes) did so for potentially serious periods. As a result, providing a fasting sample was not a requirement of the main phase of recruitment. The pilot studies also showed that a very wide range of assays could be performed in whole blood and urine samples maintained at 4°C for up to 36 hours prior to processing and storage ^9, 11^. As such, the samples were minimally processed at the assessment centres, with most of the processing conducted at the central laboratory using more efficient automated systems. The use of a standardised sample handling protocol ensured that all samples were treated in the same manner and thus will be similarly affected by any pre-analytical sample processing effects, should they exist. The processing performed immediately at the assessment centre involved inverting the plasma and serum tubes to mix the preservative/anti-coagulant with the blood (in a consistent fashion for all tubes) and then allowing the serum tube to clot at room temperature for 30 minutes. The lithium heparin and silica clot accelerator tubes contained a gel plug that formed a barrier to cellular material but allowed the plasma/serum to pass through during centrifugation (at 4°C), thereby producing sample separation. All tubes were refrigerated (with the exception of the acid citrate dextrose tube, which was held at room temperature) until the end of the day when they were wrapped with cool packs and temperature logging devices and transported to UK Biobank’s central processing and archiving facility in Stockport.

Because of the high throughput at the central laboratory (about 6,000 sample vacutainers were separated into about 25,000 aliquots every day), samples were predominantly processed using custom-designed industrial-scale automation systems that generated about 15 million 1.4 ml aliquots for the full cohort (with only a small proportion manually aliquoted). The extensive use of automation ensured that all samples were processed quickly (an average of 24 ± standard deviation of 2.5 hours between venepuncture and sample storage), with about the same delay after collection. This was achieved by ensuring that the samples were processed at the central facility in the same chronological order in which they were collected. The use of automation also allowed for a carefully controlled data trail linking each aliquot correctly to the participant from whom they were derived and facilitating rapid retrieval of specific samples (at a maximum rate of about 1,500 aliquots per day) accurately and at low cost ^10^.

### Approaches used to reduce variation at the design stage of the project

Performing cohort-wide assays (i.e. in all of the participants at the same time) facilitates good quality control by reducing measurement error and laboratory drift that might occur with the use of different assay methods, reagents and equipment in different laboratories at different times. For example, some of the best available evidence on associations of biomarkers for cardiovascular disease ^12, 13^ and cancer ^14, 15^ comes from pooled analyses of individual person data from multiple studies (since none was large enough on its own to be reliable). However, because the measurements from the individual studies were performed in different laboratories with different assays at different times, it is difficult to determine whether variations between the study-specific results reflect assay variation or real differences between study populations ^16^. Availability of a standardized panel of biomarker measures in the UK Biobank population eliminates these sources of variability and allows direct comparisons of biomarker levels to be made across the whole cohort. In addition, performing cohort-wide assays is a much more cost-effective strategy of increasing the usability of the resource at an early stage when compared with the costs of multiple retrievals and assays of samples in nested case-control comparisons based on different subsets of the participants.

### Approaches used to reduce variation during sample retrieval

In order to avoid biases in epidemiological comparisons – that is, to ensure that assay drift, reagent batch effects and other systematic measurement errors do not systematically differ between samples in subsequent case-control comparisons – samples should be assayed in as random a manner as possible (i.e. not grouped according to an underlying phenotype, date or time of blood collection, geography, etc.). However, extracting samples in a randomised sequence would have required the robotics system to access individual freezer racks multiple times, thereby substantially extended the costs and duration of the project. We therefore developed algorithms designed to select aliquots in a quasi-random sequence that avoided clustering of samples by geography and collection dates or time of day but was still efficient to implement. (For further details, please see UK Biobank Biomarker Project: Companion Document to Accompany Serum Biomarker Data).

Simulations of the performance of this picking strategy demonstrated its effectiveness and efficiency for the different sample types and ensured that participant samples were analysed in an effectively random manner throughout the assay period. This also enabled us to assess the variation caused by laboratory/assay drift, as the mean true biomarker concentration across batches and analysers should be broadly the same. In order to avoid unnecessary freeze-thaws, only tubes required for the biochemistry assays were extracted from the freezer, with the remaining aliquots on each plate returned still frozen to the working archive.

### Approaches used to reduce variation during assay measurement

Monitoring quality control is vitally important when measuring a biomarker at scale due to the number of reagent/lot changes involved, analytical machines required and duration of the assay period. We employed a series of robust and detailed quality procedures designed to minimise drift, bias and measurement uncertainty, such that each biochemistry assay result could be directly compared with another throughout the assay period (Nov 2015 to Oct 2017). In addition, in order to directly compare values between the baseline and repeat assessment sample, the repeat samples were assayed at the same time as the baseline samples and were allocated at random to assay batches.

Various immunoassay and clinical chemistry analysers were used to measure the biochemistry markers ( Table 2). During the project, the UK Biobank laboratory was successfully accredited against the internationally recognised standard for testing and calibration (ISO 17025:2005) in December 2015 for the urine and red blood cell assays, and in October 2016 for the serum assays. Each individual assay achieved a level of performance that was in agreement with the manufacturer’s claims and/or published total allowable error limits based on known biological variation.

**Table 2.** Assay instrumentation, methodology and manufacturer for each biomarker.

Serum Assay	Sample type	Analysis Methodology	Analytical Platform	Assay Manufacturer
**Alkaline Phosphatase**	serum	Enzymatic Rate	AU5800	Beckman Coulter (UK), Ltd
**Albumin**		Colourimetric		
**Alanine Aminotransferase**		Enzymatic Rate		
**Apolipoprotein A1**		Immuno-turbidimetric		
**Apolipoprotein B**		Immuno-turbidimetric		
**Aspartate Aminotransferase**		Enzymatic Rate		
**High Sensitivity C-Reactive Protein**		Immuno-turbidimetric		
**Calcium**		Colourimetric		
**Cholesterol**		Enzymatic		
**Creatinine**		Enzymatic		
**Direct Bilirubin**		Colourimetric		
**Gamma-Glutamyltransferase**		Enzymatic Rate		
**Glucose**		Enzymatic		
High Density Lipoprotein		Enzyme Immuno-inhibition		
Low Density Lipoprotein		Enzymatic Selective Protection		
**Phosphate**		Colourimetric		
**Rheumatoid Factor**		Immuno-turbidimetric		
**Total Bilirubin**		Colourimetric		
**Total Protein**		Colourimetric		
**Triglyceride**		Enzymatic		
**Urate**		Enzymatic		
**Urea**		Enzymatic		
**Lipoprotein (a)**		Immuno-turbidimetric	AU5400	Randox Bioscience, UK
**Cystatin-C**		Immuno-turbidimetric	Siemens Advia 1800	Siemens plc
**Insulin-like Growth Factor-1**		Chemiluminescent Immunoassay – one step sandwich	DiaSorin Liaison XL	Diasorin Ltd.
**Vitamin D**		Chemiluminescent Immunoassay- direct competitive		
**Oestradiol**		Chemiluminescent Immunoassay- competitive binding	Beckman Coulter DXI 800	Beckman Coulter (UK), Ltd
**Testosterone**		Chemiluminescent Immunoassay- competitive binding		
**Sex Hormone-Binding Globulin**		Chemiluminescent Immunoassay – 2 step sandwich		
**Glycated haemoglobin**	Red blood cells	High Performance Liquid Chromatography	Bio-Rad Variant II Turbo	Bio-Rad Laboratories, Inc.
**Microalbumin**	Urine	Immuno-turbidimetric	AU5400	Randox Bioscience, UK
**Enzymatic creatinine**		Enzymatic		Beckman Coulter (UK), Ltd
**Potassium**		ISE Ion Selective Electrode		
**Sodium**				

Each assay was registered with an external quality assurance (EQA) scheme and assay performance was externally verified via the results returned from participation in these schemes. We also followed a rigorous internal quality control (QC) protocol to assess precision (using different concentrations of QC samples over multiple batches and analysers) and accuracy and bias (using EQA or other commercially validated material). This involved including QC samples at the start and end of each batch such that the closing QC bracket of one batch formed the opening QC measurement of the next. We verified that the assays were linear over the observed reportable range (using commercial linearity standards and low concentration samples) and that there were no carryover effects (using low and high concentration samples analysed consecutively in a standardised sequence). We also performed multi-instrument comparisons by measuring the same sample on multiple machines of the same type and assessed potential assay interferences on each sample that could cause falsely high or low results, as detailed here: UK Biobank Biomarker Project: Companion Document to Accompany Serum Biomarker Data.

### Post-hoc analytical approaches used to reduce variation caused by unexpected dilution during sample processing at time of initial collection

During routine quality control checks, the laboratory team observed that some assay results were lower than expected for samples acquired during certain time periods. The problem was observed to increase with aliquot number and so after detection the laboratory prioritised use of aliquot 1. It appeared that, during the initial sample processing at the time of sample collection (for both the baseline and repeat assessment), some aliquots of participant serum (and plasma) samples were inadvertently diluted by the automation system during their creation from the serum vacutainer. This dilution was caused by the failure of seals to hold a system vacuum in the automated liquid handling systems so that the participant sample was mixed with system fluid (water). This issue does not affect manually aliquoted aliquots or the HbA1c results obtained from the aliquots of red blood cells (glycated haemoglobin) nor the urine measures (creatinine, microalbumin, sodium, potassium).

Consequently, at the end of the assay period, a statistical investigation was conducted to generate an initial estimation of the apparent dilution of serum assays in a given sample (using a ‘one model fits all assumption’) and to consider whether any results should be corrected or excluded. There were three main stages to the process of estimating and partial correction for the unplanned dilutions:

(i) detection of time periods with differing dilutions;
(ii) estimation of the apparent dilutions of each assay result;
(iii) evaluation of the extent of the problem and application of a correction and exclusions.

Assays are positive-valued and typically have distributions between normal and log-normal. The dilution and calibration effects would be anticipated to have approximately proportional effects across different true values. Therefore, log transformed assay values have been considered in all the correction processes.

***Stage 1: Detection of time periods with differing dilutions***. During recruitment, most serum samples were processed using 6 liquid handling machines, each with 8 tips, giving 48 machine-tip combinations. The blood samples collected at the repeat assessment visit were processed using 5 liquid-handling machines. A single tip was used to dispense up to 4 aliquots from a participant sample. A small proportion of samples from each assessment were manually aliquoted. Results were reported as coming from aliquot 1–4, a manually generated aliquot, or this information was missing. Only aliquots 1–3 have been included in the dilution estimation process as there were not enough sample results from aliquot 4s for accurate assessment of the dilution problems.

The machine-tips may have operated with undetected faulty seals for several days. Further, upon detecting a faulty seal, the seal may not have been immediately replaced, in part due to a lack of replacement parts. Consequently, it should be possible to identify periods with unexpected dilutions on particular machine-tips by looking at assay results in the order in which they were collected. The precise time of aliquot generation is not available, but in general the aliquots were generated within 24 hours of participant sample collection. Therefore, the date of the participant attending the assessment centre is a reasonable proxy for this information, with a 1 day resolution.

Variable dilutions would not only affect the mean assay values but would also contribute artefactual correlation between the results from different assays on the same sample. The magnitude of this artefactual correlation would, in principle, depend on the true correlation and the extent to which the two assays were affected by dilution. In practice, the apparent dilutions observed may also be influenced by ‘matrix’ effects, whereby on dilution by a given percentage with water, different assays do not perform in an entirely *pro rata* manner. Such ‘matrix’ effects may be particularly likely for assays where the specified diluent (for assaying high values) is not water: this includes 11 of the assays (testosterone, oestradiol, vitamin D, insulin-like growth factor-1, sex hormone-binding globulin, lipoprotein (a), C-reactive protein, rheumatoid factor, apoliporotein A apolipoprotein B, cystatin C).

After some exploratory investigations, 7 assays (albumin, calcium, creatinine, cystatin C, glucose, phosphate, and total protein), selected from among those most strongly affected by dilution by various criteria, were used in the analysis to detect time periods with different dilutions. A multivariate change point analysis was conducted using the selected 7 assays to identify, for each machine-tip, time points (of sample collection) at which there were jumps in mean assay levels.

Non-reportable assay values below or above the reportability limits were included in the change point analysis but not in later stages of analysis and are set to missing in the dataset.

**Change point analysis**

• Performed in 7 selected assays (those most strongly affected by dilution by various criteria) and restricted to samples with complete data on aliquot number and machine-tip;

• Means of the log-transformed values for each machine-tip by collection day were generated;

• Each day was treated as a 7-dimensional observation from a multivariate time series using the ecp algorithm in R 3.5.0 to identify significant change points with the minimum time period set at 3 days. This procedure uses permutation testing (10,000 repeats) to identify significant changes in the distribution and a P-value of 0.01 was used;

• The algorithm was run independently for each machine-tip (48 at recruitment + 40 at repeat visit) x aliquot number (3);

• After running the above analyses, to consolidate the change points for each machine-tip, change points occurring in any aliquot within 3 days of each other were consolidated as a single change point (with the earliest date allocated); in addition, periods were merged with adjacent periods where necessary to have a minimum of 30 samples per period.

**Figure 1.**
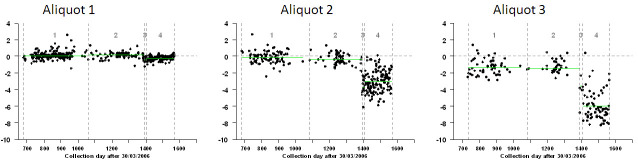
Example of change-points identified across the aliquots for a specific machine-tip during one of the worst affected periods.

The change point analysis divided the sample collection timeframe for each machine-tip into periods with distinct assay performance, as shown in Figure 1. This process brought to light a few periods with other anomalies requiring removal of the samples and re-running the change point analysis.

**Issues identified: ‘Dips’ and aliquot misclassifications** Some time periods showed a few other obvious anomalies likely to correspond to severe dilution problems or aliquot number misclassification.

**Dips** Visual assessment of some of the assay results identified short periods of time on particular machine-tips when the values were highly variable, even in aliquot 1. We refer to such periods ‘dips’, since the results visually dip very low compared to the overall population. As this could be an indication of a severe dilution problem, results from these samples were excluded from the final estimation and from both the main and extended dataset. 

**Possible aliquot number misclassification** Visual inspection of some of the assay results also identified periods on particular machine-tips when assay results appeared to have a bimodal distribution (with the lower valued component being more out of line), suggesting possible aliquot misclassification, meaning that one of the populations of results may have come from a different aliquot to that recorded by the lab. We would expect the proportion of samples from each aliquot to be similar across the tips within a machine, but checking this identified 4 periods with different proportions of results from each aliquot and with a bimodal distribution suggesting they were two distinct populations of results. This was indicative of possible aliquot misclassification.

Where aliquot misclassification was suspected, the mclust algorithm (implemented in the R 3.5.0 package *mclust*) was used to generate estimates for the means and standard deviations (SDs) of a 2 component mixture of Normal distributions. This procedure maximises the Bayesian Information Criteria (BIC) for the mixture, allowing different means and different SDs in the 2 mixtures. The split point, x _s_, where there is the same probability that a value belongs to each mixture component is estimated. We reject results from participants with results below x _s_, (i.e., estimated to be from the component with the lower mean) suspecting aliquot misclassification.

**Outcome of the change point analysis** The process identified change points in nearly all machine-tips. In the baseline sample, the process identified a total of 103, 2, and 1 change points from the analysis of aliquots, 1, 2 and 3, respectively, giving 106 detected change points in total. Of the 48 machine-tips, 3 had no change points, 6 a single change point and 39 had two or more change points.

***Stage 2: Estimation of dilution***. Details of the inclusion criteria applied for the dilution estimation are provided in the extended data ^17^. In brief, assay results were excluded if the aliquot had failed by the processes described above, or aliquot 4 was used, or if the analyser provided an unreportable result. Assays excluded from dilution estimation included those with a high biological coefficient of variation (lipoprotein (a), gamma glutamyltransferase, C-reactive protein, rheumatoid factor) plus vitamin D, where seasonal variation masked any dilution effects related to time of sample acquisition, leaving 24 assays included.

Estimates of dilution were only performed in the serum samples. The single assay performed in red blood cells (HbA1c) was not considered in the estimation of dilution as any effect of dilution is unlikely to affect the results, given that HbA1c is presented as a concentration ratio. Additionally, only 1 aliquot was dispensed (from 2 EDTA sample tubes), and hence it is not possible to assess the effect of dilution across different aliquots. As such, the HbA1c assay results do not have a corresponding aliquot number associated with them. There also appears to be no dilution issue for the 4 assays performed in urine.

**Modelling dilution** For each assay result the dilution factor was defined as the multiplicative factor applied to the theoretical true result (i.e. from perfect aliquoting) which would give the observed result, i.e. a dilution factor of 85% means observed=0.85×theoretical true (so 1/0.85≈1.17 means that the system fluid represented a 17% additional volume).

The basic principle of the correction is that, for an observed assay result Y _diluted_

Y _diluted_ = dilution_factor × Y _true_ + error

After taking logarithms of the assay results, the multiplicative factor becomes an additive factor relative to manual aliquots, and the model becomes

log(Y _diluted_) = log(Y _true_) + log(dilution_factor) + E, E~N(0,SD ^2^)

We anticipate that the dilution factor depends on aliquot×machine_tip_period, but in addition, the apparent dilutions observed for different assays will vary somewhat around the actual sample dilution due to substrate ‘matrix’ effects, whereby different assays do not yield completely pro rata effects for a given dilution with water. Therefore, the model fitted also includes a term for assay×aliquot. As this method is only applicable to variables with equal variance, the terms are divided by the SD of the log assay. Testosterone and oestradiol were included as separate assays for each sex, taking the number of ‘assay’ categories in the model from 24 to 26. Hence the model fitted was

LYS _ij_ = assay _j_ + assay _j_×aliquot _i_ + aliquot _i_×machine_tip_period _i_

where:

- LYS _ij_ is the log assay result for assay j (j=1,… 26) for sample i divided by the standard deviation of the log results for that assay
- assay _j_ is a categorical variable for the assay (j=1,…26) yielding result Y _ij_
- aliquot _i_ is a categorical variable for the aliquot number (0–3, where 0 denotes manually aliquoted) for sample i
- machine_tip_period is a categorical variable for the machine tip used for sample i

Separate models were fitted to the baseline and repeat visit results (as the machine-tip-periods were distinct). The parameter estimates were calculated with manual aliquots (~9,000 of the baseline samples and ~6,000 of the repeat assessment samples) as the reference group, but in a further step were referenced to a larger group by the addition of a constant to yield an average dilution factor of 1 in the larger group (defined as participants with manual aliquots plus samples with calcium results from aliquot 1 with an estimated dilution factor of 0.99–1.01: this group contained ~350,000 samples at baseline).

Since LYS _ij _is log transformed, we can interpret the coefficients as a scaling applied to the original untransformed variables. Therefore, for a given assay result from a given aliquot and machine_time_period, if we estimate β as the assay _j_×aliquot effect and δ as the aliquot×machine_time_period effect, then exp(β)×exp(δ) = exp(β+δ) is the estimated apparent dilution factor.

The estimated sample dilution factor was calculated as the exponential of a weighted average with weights 1/SD _j_^2^ of the model terms relevant to the dilution in each assay over 17 assays, excluding assays with a significant proportion of results below the lower reportable limit and assays where the normal diluent was not water) i.e., for sample i

exp(∑ _j=1, 17_ (assay _j_×aliquot _i _and aliquot _i_×machine_tip_period _i_) /SD _j_^2 ^x ∑ _j=1,17_ SD _j_^2^)

We refer to (1-estimated sample dilution factor) x 100 as the estimated percentage reduction in sample concentration.

The model parameters were estimated using PROC GLM in SAS 9.4. 10-fold cross-validation was used to avoid overfitting (i.e. the samples were randomly assigned into 10 groups; for each 10 ^th^, the other 90% of the data was used to generate parameter estimates for that 10th of the data).

***Stage 3: Evaluation and decisions on correction and exclusion from the UK Biobank Data Showcase***. After excluding assays with a significant proportion of results below their lower limit, the extent of the dilution problem was characterised for the remaining assays included in the dilution estimation by comparing the correlations between assays in manually aliquoted samples with those in samples from a given aliquot number or a given estimated sample dilution range. These differences in correlations were reviewed using heatmap visualisations (data not shown). Four assays with the lowest biological CVs (calcium, total protein, phosphate and albumin) showed distorted correlations with each other and to a lesser extent with some other assays. In addition, a further 4 assays (glucose, high-density lipoprotein, apolipoprotein (a), sex hormone-binding globulin) also showed substantially distorted correlations with several assays. The distortions tended to increase with aliquot number and with estimated reduction in sample concentration and with estimated reduction in sample concentration within a given aliquot number, where this was assessable. Therefore, the estimated sample reduction in concentration appeared to add some information on dilution over and above aliquot number, at least in these ranges, and so the full model was adopted as the method of estimating the sample dilution.

However, applying the modelled predictors of the apparent assay result dilution factors (involving about 500 terms) to assay results to correct for dilution, made only a small improvement to the correlation distortions This failure may be partly because only 3 of the change points identified were in aliquots 2 and 3, which was probably primarily due to low frequency of use of these aliquots. However, it could also be an indication that the dilutions were varying over a shorter timeframe than could be captured by the present model and data. The majority of the change points were derived in aliquot 1, where the dilutions were small and any improvement from correction would be largely negligible and difficult to evaluate. It is also possible that differences between assays may not have been adequately catered for in this first pass model. Given the limited improvement achieved, the model was rejected as a satisfactory correction for assay results. Instead a further model involving just terms for assay x aliquot number was fitted to correct for differences in apparent dilutions by aliquot number (involving 3 terms per assay). The aliquot-number-corrected result was obtained by dividing the observed result by the estimated apparent dilution factor from this model based on aliquot number.

After this correction, substantial artefactual correlations between some pairs of assays remained at higher dilutions. Therefore, in addition to the exclusion criteria described for Stage 2 (and described in more detail in the extended data ^17^), results from all assays were excluded from the main dataset for samples with estimated reductions in sample concentration outside -10 to 10%, and from the 8 assays mentioned above (that showed the worst distortions in the correlations between assays) for samples with estimated reductions in sample concentration outside -1 to 1%. 

### Post-hoc analytical approaches used to reduce variation caused by laboratory drift

For each assay, plots of the daily mean results by date-of-assay were performed to investigate drifts of assay values over time. Based on this, the results from all serum assays except for vitamin D, lipoprotein (a), gamma glutamyltransferase, C-reactive protein and rheumatoid factor were corrected for date of assay effects. The date of assay correction process was also applied to HbA1c results, which were from a different type of sample (red blood cells) not investigated for dilution, but which were affected by day-to-day lab variability. The date of assay effects were assumed to be independent of the aliquot dilution problem and the correction was applied after correction for aliquot dilutions.

Least square means (LSMEANs) were generated from a linear model of log assay values (using aliquot-dilution corrected assay results, where available) on date-of-assay as a categorical variable, with adjustment for the interaction of sex with age at survey (as a categorical variable for single year [recruitment ages <40 or >70 were combined into groups for age 40 or 70, respectively]). Age and sex were included as a precaution to allay concerns that differences in the participant characteristics of the samples assayed each day might be contributing importantly to the date-of-assay effects (but the age and sex terms appeared to account for little of the day-to-day variation.) The date-of-assay effect was calculated as the difference of the date-of-assay LSMEAN from the overall mean. Values were corrected by subtracting the corresponding date-of-assay effect (resulting in correction for date of assay but not adjustment for age and sex).

Baseline and repeat assessment samples were included together in the models for each assay, as these samples were mixed together across assay batches throughout the assay period. Days with <30 observations were grouped with the neighbouring day (forwards or backwards). This was done recursively until all day groups had at least 30 observations. For each assay, the change in within-participant correlation (between recruitment and repeat assessment) with adjustment for date-of-assay was plotted against the assay biological CV to determine whether adjustment for date-of-assay improved the self-correlation.

## Results

### Managing values below the lower reportable range

All of the participant and quality control data generated were reviewed during the project to identify and address any issues in real time, and to allow retrospective adjustments to be made (where required). We excluded results where no data or error values were returned from the analyser, there was an aliquot problem, or the values were outside the reportable range of the assay at the time of measurement (and hence considered not to be sufficiently accurate or precise). Overall, this affected 9% of assay results, although some assays were more affected than others. For example, 80% of oestradiol values were below the lower reportable range (and hence excluded), which is to be expected given the age and sex range of the participants (as relatively few women were premenopausal at recruitment, and approximately half were men) and the low analytical sensitivity of the assay. The majority (91%) of values for rheumatoid factor were also below the reportable range since only individuals with (or at high risk for) rheumatoid arthritis have demonstrably measurable values ( Table 3). Hence, researchers may wish to consider these values as ‘naturally low’ rather than ‘missing’ in order to maximise the scientific utility of these data.

**Table 3.** Numbers of results below the reportable range for assays with >0.1% of results below the reportable range in baseline samples ^1^.

Aliquot	Number of original baseline results below reportable range				
	Lipoprotein A	Oestradiol (females)	Rheumatoid Factor	Testosterone (males)	Vitamin D
Manual	907 (10.5%)	3378 (75.5%)	8247 (90.7%)	7 (0.2%)	76 (0.9%)
Aliquot 1	41822 (10.3%)	157625 (75.2%)	381003 (90.6%)	184 (0.1%)	2302 (0.6%)
Aliquot 2	2921 (10.2%)	12017 (74.5%)	26751 (91.2%)	11 (0.1%)	156 (0.6%)
Aliquot 3	1079 (11.1%)	5311 (74.5%)	9798 (91.5%)	7 (0.2%)	53 (0.5%)

^1^ No assays had >0.1% of results above their reportable range.

### Correction for unexpected dilution during sample processing at time of initial collection

The model incorporating aliquot-number specific shifts in dilutions over time of aliquoting provided an indication of the extent to which each sample was affected by dilution ( Table 4).

**Table 4.** Distribution of estimated percentage reductions in sample concentration by aliquot number ^1^.

Aliquot Number	Number of samples	Estimated percentage reduction in serum sample concentration							
		≤-2% ^2^	>-2% to ≤-1% ^2^	>-1% to <-1%	≥1% to <3%	≥3% to <5%	≥5% to <10%	≥10% ^3^	Mean
Manual	9,086	-	-	100%	-	-	-	-	-
1	418,170	0.00%	0.21%	98.54%	1.25%	-	-	-	0.1%
2	29,050	-	0.01%	20.55%	77.88%	1.56%	-	-	1.4%
3	10,230	0.01%	-	-	0.02%	1.94%	97.97%	0.07%	6.4%
Total	466,536	0.00%	0.19%	91.55%	5.97%	0.14%	2.15%	0.00%	0.3%

^1^ Assays excluded from the dilution estimation included those with a high biological coefficient of variation (CV) (lipoprotein (a), gamma glutamyltransferase, C-reactive protein, rheumatoid factor) plus vitamin D, where seasonal variation masked any dilution effects related to time of sample acquisition, leaving 24 assays included. Also excluded from this table were participant samples where the aliquot number used varied between assays (see the Extended Data for further details). ^2^ A small number of samples had estimated concentrations higher than the theoretical true result (i.e. derived from perfect aliquoting). ^3^ Results for serum assays with estimated percentage reduction in sample concentration ≥10% were set to missing.

The dilution is partially systematic in that the magnitude of the dilution increases with increasing aliquot number (i.e. aliquot 1 is less affected than aliquot 2, etc.). Because a concern was raised soon after the assays started, the laboratory quickly prioritised use of aliquot 1 in order to reduce the impact of the dilution on the results.

Overall, 98.5% of assay results from aliquot 1 (which accounts for 90% of participant serum samples) have an estimated concentration that is less than 1% different to that of the theoretical true result (i.e. derived from perfect aliquoting), and 1.5% have an estimated concentration up to 3% different. For aliquot 2, most sample reductions in concentration are in the range 1–3%. Aliquot 3 is more affected by dilution, although this only accounts for a small proportion (2%) of samples that have been assayed. Here, almost all of the samples are diluted in the range of 5–10%, with only 7 samples (0.07%) having a greater estimated reduction in concentration ( Table 4). All results from aliquot 4 were excluded as there were not enough results for accurate assessment of the dilution problem in them. 

Assays with a naturally wide biological range are typically far less materially affected from an epidemiological perspective by a given dilution, as small dilution errors are small compared with the biological variation across the population. Conversely, assays with a narrow biological range (e.g., albumin, calcium, phosphate, total protein) are more materially affected. For this reason, we excluded results with estimated differences in sample concentration greater than 1% for these particular analytes, as well as for 4 further analytes (apolipoprotein (a), glucose, high-density lipoprotein, sex hormone-binding globulin) that appeared importantly affected. For all other assays, we excluded results if the estimated sample reduction in concentration was greater than 10% (which affected 7 samples, all from aliquot 3).

Because the extent of dilution is strongly related to aliquot number, adjustment of the assay results for aliquot number provides a simple ‘first-pass’ approach to dealing with this issue (coupled with exclusion of assay results deemed to be materially affected by dilution).

In order to improve confidence in the accuracy and precision of the aliquot-adjusted result, we have only included the results in the main dataset (available in the core dataset of the Data Showcase) if the assay value was within the reportable range before and after aliquot adjustment. However, an extended dataset is available (via the Return of Results catalogue) with all the adjusted results, including those that are outside the reportable range after adjustment (but were reportable originally), with a flag indicating if the value was rejected or not in the main dataset, as it is possible that those results may nonetheless prove useful for epidemiological purposes.

### Correcting for expected laboratory drift of assay results

Owing to the random selection of the participant samples and sheer volume of results, it was possible to perform statistical analysis of the participant data to ensure that the day-to-day variation was within acceptable limits, as the overall mean of the biomarker levels should not vary across batches over time owing to the random plating of samples. Although analytical variation was minimised using rigorous internal QC checks, plots of the daily mean results by date of assay revealed that some assays exhibited large amounts of daily variability. Changes in reagent batches could account for some of the variation but other variation was detectable by the volume of results in comparison to the limitations of standard lab QC procedures.

**Figure 2.**
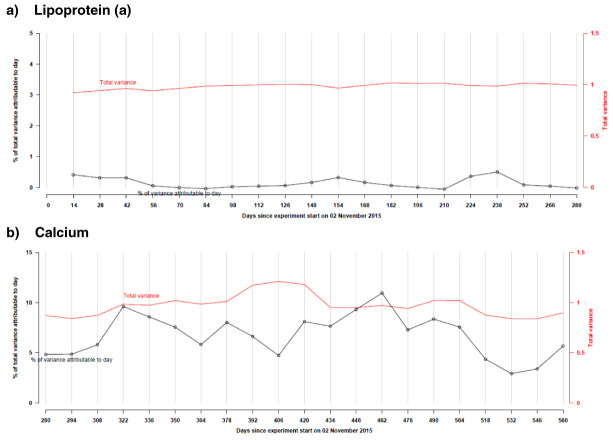
Total variance (right-hand y-axis) and proportion of total variance explained by date of assay (left-hand y-axis) over selected periods of time for **a**) lipoprotein ( **a**) and **b**) calcium, both in standardised units.

The impact of this variation on epidemiological analyses depends on the biological variation of the assay. For assays with high inter-individual variability, such day-to-day variation accounts for a small proportion of the total variation (e.g., about 0.5% for lipoprotein (a) and C-reactive protein); conversely, for biomarkers that are more tightly regulated, this laboratory variation may be more material (e.g. about 10% for calcium; Figure 2).

In order to correct for laboratory variation over the assay period (caused by changes in reagent batches, etc.), all assay results were adjusted for date of assay, with the exception of vitamin D, lipoprotein (a), gamma glutamyltransferase, C-reactive protein and rheumatoid factor. These five assays have a high biological coefficient of variation or evident seasonal variability (as is the case for vitamin D), and correction for date of assay did not improve the within-participant correlation between the baseline and repeat assessment samples, presented in Table 5 (used in this context as an additional marker of data quality). In addition, for the four urine assays (sodium, potassium, microalbumin, and creatinine), we did not correct for date of assay as day-to-day variation only accounted for a minimal proportion (<2%) of the total variation in assay values.

**Table 5.** Mean values from the baseline and repeat assessment sample according to fourths of the biomarker distribution from the baseline sample, and their self-correlation for all assays ^1^.

	Baseline measures in fourths				Range of means across baseline categories	Correlation between baseline and repeat measure
**Alanine aminotransferase** **[ALT] (U/L, n=16,610)**	**1**	**2**	**3**	**4**		
Baseline sample mean	12.8	17.9	23.4	39.5	26.7	0.46
Repeat sample mean	16.4	20.1	23.9	31.5	15.1	
**Albumin [ALB] (g/L,** **n=13,434)**	**1**	**2**	**3**	**4**		
Baseline sample mean	41.9	44.3	45.9	48.3	6.4	0.48
Repeat sample mean	44.0	45.3	46.1	47.2	3.2	
**Alkaline phosphatase** **[ALP] (U/L, n=16,616)**	**1**	**2**	**3**	**4**		
Baseline sample mean	57.2	73.2	86.3	112.3	55.2	0.72
Repeat sample mean	64.4	77.7	88.5	107.9	43.5	
**Apolipoprotein A** **[APOA] (g/L, n=13,290)**	**1**	**2**	**3**	**4**		
Baseline sample mean	1.2	1.4	1.6	1.9	0.7	0.77
Repeat sample mean	1.3	1.5	1.6	1.9	0.5	
**Apolipoprotein B** **[APOB] (g/L, n=16,485)**	**1**	**2**	**3**	**4**		
Baseline sample mean	0.7	0.9	1.1	1.3	0.6	0.66
Repeat sample mean	0.8	1.0	1.1	1.2	0.4	
**Aspartate aminotransferase** **[AST] (U/L, n=16,495)**	**1**	**2**	**3**	**4**		
Baseline sample mean	18.7	22.9	26.6	36.4	17.7	0.40
Repeat sample mean	21.8	24.7	27.4	32.5	10.7	
**C-reactive protein** **[CRP] (mg/L, n=16,551)**	**1**	**2**	**3**	**4**		
Baseline sample mean	0.4	0.9	1.7	6.3	6.0	0.29
Repeat sample mean	1.0	1.7	2.3	4.4	3.4	
**Calcium [CA] (mmol/L,** **n=13,431)**	**1**	**2**	**3**	**4**		
Baseline sample mean	2.3	2.3	2.4	2.5	0.2	0.42
Repeat sample mean	2.4	2.4	2.4	2.5	0.1	
**Cholesterol** **[CHOL] (mmol/L, n=16,623)**	**1**	**2**	**3**	**4**		
Baseline sample mean	4.3	5.3	6.0	7.1	2.9	0.66
Repeat sample mean	4.7	5.5	6.0	6.7	2.0	
**Creatinine [CRE] (umol/L,** **n=16,580)**	**1**	**2**	**3**	**4**		
Baseline sample mean	55.7	66.7	76.2	91.7	36.0	0.77
Repeat sample mean	59.9	69.7	78.8	91.7	31.8	
**Cystatin C [CYS] (mg/L,** **n=16,608)**	**1**	**2**	**3**	**4**		
Baseline sample mean	0.7	0.8	0.9	1.1	0.3	0.81
Repeat sample mean	0.8	0.9	0.9	1.1	0.3	
**Direct bilirubin** **[BILD] (umol/L, n=12,635)**	**1**	**2**	**3**	**4**		
Baseline sample mean	1.2	1.5	1.9	3.0	1.8	0.70
Repeat sample mean	1.4	1.6	1.8	2.6	1.2	
**Gamma glutamyltransferase** **[GGT] (U/L, n=16,604)**	**1**	**2**	**3**	**4**		
Baseline sample mean	14.9	21.9	31.6	72.6	57.8	0.67
Repeat sample mean	17.1	24.3	34.0	64.9	47.8	
**Glucose [GLU] (mmol/L,** **n=13,411)**	**1**	**2**	**3**	**4**		
Baseline sample mean	4.2	4.7	5.1	6.1	1.9	0.42
Repeat sample mean	4.8	4.9	5.0	5.5	0.7	
**HbA1c (mmol/mol, n=12,863)**	**1**	**2**	**3**	**4**		
Baseline sample mean	30.5	34.0	36.3	42.2	11.7	0.76
Repeat sample mean	32.5	35.0	36.7	42.0	9.4	
**HDL cholesterol** **[HDLC] (mmol/L, n=13,430)**	**1**	**2**	**3**	**4**		
Baseline sample mean	1.0	1.3	1.5	2.0	0.9	0.85
Repeat sample mean	1.1	1.4	1.6	2.0	0.8	
**Insulin-like growth factor-1** **[IGF-1] (nmol/L, n=16,357)**	**1**	**2**	**3**	**4**		
Baseline sample mean	14.9	19.7	23.1	28.6	13.7	0.77
Repeat sample mean	15.7	19.5	22.2	26.3	10.6	
**LDL direct** **[LDLD] (mmol/L, n=16,538)**	**1**	**2**	**3**	**4**		
Baseline sample mean	2.5	3.2	3.8	4.7	2.2	0.65
Repeat sample mean	2.8	3.4	3.8	4.3	1.5	
**Lipoprotein (a)** **[LPA] (nmol/L,** **n=12,203)**	**1**	**2**	**3**	**4**		
Baseline sample mean	6.7	14.4	35.3	112.2	105.4	0.95
Repeat sample mean	8.2	16.6	37.3	116.6	108.4	
**Oestradiol** **[OES] (women only; pmol/L,** **n=659)**	**1**	**2**	**3**	**4**		
Baseline sample mean	227.0	345.9	517.8	1116.3	889.3	0.07
Repeat sample mean	518.7	526.7	612.2	666.5	147.8	
**Phosphate** **[PHOS] (mmol/L, n=13,378)**	**1**	**2**	**3**	**4**		
Mean in baseline sample	1.0	1.1	1.2	1.4	0.4	0.45
Repeat sample mean	1.1	1.2	1.3	1.3	0.2	
**Rheumatoid factor** **[RF] (IU/mL, n=1,009)**	**1**	**2**	**3**	**4**		
Baseline sample mean	11.8	16.6	25.8	53.6	41.9	0.58
Repeat sample mean	17.8	20.0	27.6	45.9	28.1	
**Sex hormone-binding** **globulin** **[SHBG] (nmol/L, n=13,130)**	**1**	**2**	**3**	**4**		
Baseline sample mean	25.0	39.0	53.4	87.4	62.4	0.81
Repeat sample mean	30.3	43.6	57.5	83.5	53.2	
**Testosterone** **[TES] (men only; nmol/L,** **n=8,124)**	**1**	**2**	**3**	**4**		
Baseline sample mean	7.9	10.8	12.9	16.8	8.9	0.66
Repeat sample mean	9.3	11.3	12.8	15.5	6.2	
**Total bilirubin** **[TBIL] (umol/L, n=16,452)**	**1**	**2**	**3**	**4**		
Baseline sample mean	5.5	7.4	9.2	14.9	9.4	0.77
Repeat sample mean	6.4	7.5	8.7	13.2	6.8	
**Total protein [TP] (g/L,** **n=13,420)**	**1**	**2**	**3**	**4**		
Baseline sample mean	67.2	70.6	73.0	77.0	9.9	0.50
Repeat sample mean	70.6	72.6	74.0	75.9	5.3	
**Triglycerides** **[TRIG] (mmol/L, n=16,585)**	**1**	**2**	**3**	**4**		
Baseline sample mean	0.8	1.2	1.7	3.0	2.2	0.60
Repeat sample mean	1.1	1.4	1.8	2.5	1.4	
**Urate [UA] (umol/L,** **n=16,560)**	**1**	**2**	**3**	**4**		
Baseline sample mean	213.4	277.3	329.6	410.9	197.5	0.82
Repeat sample mean	231.9	288.8	336.2	399.6	167.7	
**Urea (mmol/L, n=16,587)**	**1**	**2**	**3**	**4**		
Baseline sample mean	4.0	5.0	5.7	7.1	3.2	0.59
Repeat sample mean	4.7	5.3	5.8	6.6	1.9	
**Vitamin D [VITD] (nmol/L,** **n=15,437)**	**1**	**2**	**3**	**4**		
Baseline sample mean	25.4	41.1	55.2	76.8	51.4	0.56
Repeat sample mean	34.0	42.3	50.4	64.3	30.3	

^1^ Includes biochemistry markers measured in serum and red blood cells (HbA1c).

### Regression dilution bias

Due to the combined effects of measurement error and within-person biological variability over time, single baseline measurements of biomarkers do not, on average, reflect ‘usual’ medium-term levels, leading to a systematic underestimation of the strength of the association of biomarkers with other factors when baseline levels are used in analyses ^2, 18^. To allow correction for this ‘regression dilution bias’, we performed the biochemistry assays on blood samples taken from 20,000 participants who attended a repeat assessment visit approximately 4–5 years after their baseline visit. Almost all biochemistry values show some regression to the mean, predominantly observed in the top and bottom baseline categories, although the magnitude of this change varied by biomarker. For example, oestradiol and glucose showed the highest regression dilution bias, with the range of mean values from the repeat assessment sample being substantially narrower than that from the baseline sample, which is also reflected in the low self-correlation between the two measures ( Table 5). This likely reflects true biological within-person variability owing to oestrogen fluctuations across the menstrual cycle and transition through the menopause during follow-up, and glucose fluctuations by time since last meal. In contrast, HDL-cholesterol and lipoprotein (a) had consistent mean values between baseline and repeat measures and a high self-correlation ( Table 5).

Correction of association analyses for regression dilution bias can be performed using standard statistical approaches ^2, 18, 19^. A non-parametric approach that is commonly used estimates the regression dilution ratio as the ratio of the differences between repeat and baseline measures across equal groups defined by the baseline measure ( Table 5; ^18^). Examples of alternative parametric methods include dividing the beta coefficient (and its standard error) of the association by the regression dilution ratio, as estimated either by the correlation ( *r*) between baseline and repeat measures ( Table 5; ^20^) or by a linear regression of the repeat assessment values on the baseline measures ^21^.

## Conclusions

The availability of data on a wide range of key biochemistry markers for all 500,000 participants, measured in a highly standardised and systematic manner are a valuable enhancement to UK Biobank, as they enable comparisons of biomarker results across the entire cohort (e.g., between cases of any given health outcome of interest and non-cases). Hence, we envisage that these data will enable researchers to investigate the development of a wide range of health outcomes using both traditional and novel analytical strategies.

### Epidemiological considerations for the analysis of the biochemistry data

In order to help minimise the impact of measurement error in assay data for a very large number of samples, it is important to apply robust QC procedures and to identify and mitigate variations that arise. During the assay of 34 biochemistry markers for 500,000 participants in UK Biobank, we identified and applied adjustments for a number of sources of variation.

### Use of repeated measures

The availability of repeat biochemistry measures in UK Biobank allows researchers to account for regression dilution bias, caused by measurement error and/or true biological variability in values over time. The observation that some of the biochemistry markers showed substantial regression to the mean when comparing the values taken from a repeat assessment sample with that from the baseline sample suggests that failure to consider such regression dilution bias in analyses will underestimate any associations found between biomarkers and other factors. In addition to the repeat assessment samples collected in 20,000 participants approximately 4–5 years after recruitment, UK Biobank is also collecting blood samples from 100,000 participants who are undertaking an imaging assessment between 7 and 12 years after recruitment, which will allow further examination of random variation and intra-individual changes over time, should these biochemistry markers be measured again.

### Managing sample aliquoting variation caused by unexpected dilution

The adjustments that have been made for the unexpected dilution of some aliquots are a first-pass approach and may have limitations. Some analytes are affected more than others, so researchers should be cognisant of the size of the adjustment applied to biomarkers of interest. Open-access nature to the original values and laboratory parameters allows further investigation of this issue by other researchers. This will provide the opportunity for researchers to scrutinise the applicability of the adjustment across the whole range for particular analytes and to propose enhanced adjustments and/or guidance on which samples to include for specific analyses. For example, the analyses did not identify an entirely consistent pattern of variation across different analytes. Researchers with expertise in particular assays or more advanced modelling may be able to improve the adjusted data in the UK Biobank resource (including, for example, better characterisation of the variation in the excluded results, with a view to potentially retrieving some of these results).

92% of these assays were performed in aliquot 1 (because the laboratory team preferentially selected it when the issue was first identified) or the manually aliquoted samples. Researchers could consider performing sensitivity analyses (e.g. comparing the results of analyses before and after restricting to results derived from aliquot 1) or stratifying the analyses by aliquot number to assess the potential impact of dilution (and its adjustment) on the interpretation of epidemiological results. Particular consideration is needed for analyses that include results from several assays, as the errors associated with any dilution effect will be the same for all of the analytes assayed in the same aliquot and, even if small, may result in artefactual positive correlations.

For future assays, such as metabolomics or proteomics, UK Biobank will carefully consider which aliquots to use. For example, assays that are semi-quantitative in nature, have a naturally wide biological range or require dilution prior to measurement, are unlikely to be unduly affected by a small amount of variation caused by dilution. The choice of aliquot number to use and the need for sensitivity analyses may also depend on the nature of the research question. For example, in analyses that focus on the association of an analyte with other measures (e.g. a genetic variant or disease incidence), such variation in the assay values will be random and the additional power from using data from all of the samples is likely to outweigh such random variation. Performing future assays in the same aliquot (e.g., aliquot 3) for all samples should help to mitigate the systematic error associated with increasing amounts of dilution across aliquot number (although a downward bias in the mean levels, as well as some residual random variation, is likely to persist). As such, we would advise researchers conducting future assays to include a small number of samples using aliquot 1 or manual aliquots, or to perform their own dilution experiments, to examine the extent to which dilution affects the analytes of interest.

In summary, UK Biobank has adopted several approaches to ensure that the data generated from assays of 34 biochemistry markers for all 500,000 participants are suitably comparable across the entire cohort. These approaches have included careful consideration of our methods used for sample collection, processing, retrieval, assay measurement and data analysis in order to mitigate the impact of both systematic and random variation in epidemiological analyses.

### Ethics

UK Biobank received ethical approval from the National Health Service North West Centre for Research Ethics Committee (Ref: 11/NW/ 0382) and from the Human Tissue Authority. Such approvals mean that researchers wishing to use the resource do not need separate ethics approval (unless re-contact with participants is required).

## Data availability

### Underlying data

Access to UK Biobank data is available to registered researchers worldwide from across academia and industry, without the need for collaboration, to perform health-related research that is in the public interest. The main dataset containing the pre-corrected and corrected (if appropriate) results within the reportable range is located in UK Biobank’s Data Showcase in category 100079 ( http://biobank.ndph.ox.ac.uk/showcase/label.cgi?id=100079). The ‘extended’ dataset containing the pre-corrected and corrected values for all participants (i.e. including those that were outside reportable range after correction) is located as Return 1602 ( http://biobank.ndph.ox.ac.uk/showcase/dset.cgi?id=1602). Both datasets can be requested as part of an application submission (or extension).

### Extended data

Access to the extended data (that includes further details of the inclusion criteria), plus the underlying SAS code and macros used for the changepoint analysis and date of assay corrections are available in the Zenodo repository. R can be used as an alternative to SAS to estimate the model parameters.

Zenodo: Extended data: Statistical investigation of the UK Biobank biochemistry assays quality procedures. http://doi.org/10.5281/zenodo.4022776 ^17^

This project contains the following extended data:

- Extended data. Statistical investigation of the UK Biobank biochemistry assays quality procedures: includes details of the inclusion criteria for the analyses undertaken.
- adjacent_om.sas: SAS macro for calculating least square means (LSMEANS) of a dependent variable by levels of a group variable, using a linear model possibly with adjustment, and centralising estimated LSMEANS around overall mean of the dependent variable.
- apply_adjustment 201902.sas: main SAS program for calculating and applying the date-of-assay adjustment factors.
- apply_correction 201902.sas: main SAS program for calculating and applying the aliquot adjustment factors.
- crosstab.sas: main SAS program for calculating cross-tabulations of biomarkers.
- ecp_changepoints.SP.r: main R program for detecting changepoints by aliquot.
- export_csv.sas: SAS macro for saving a SAS table as a CSV file.
- general_macros.sas: helper SAS macros for loading and saving the biomarker data.
- make_dataset.sas: helper SAS macro for merging together datasets
- make_reshaped.sas: main SAS program that assembles the lab data into "long" (i.e. multiple rows per participant) format ready for the aliquot correction factors to be computed in apply_adjustment 201902.sas
- Znorm.sas: SAS macro for standardising variables by subtracting mean and dividing by standard deviation.

Data are available under the terms of the Creative Commons Zero "No rights reserved" data waiver (CC0 1.0 Public domain dedication).
