# Supplementary material for: Approaches to minimising the epidemiological impact of sources of systematic and random variation that may affect biochemistry assay data in UK Biobank
Source: Wellcome Open Res. 2021 Jan 4;5:222. Originally published 2020 Sep 24. [Version 2] doi: 10.12688/wellcomeopenres.16171.2 (PMC7739095; doi:10.12688/wellcomeopenres.16171.2)
Supplement: Peer review history

# Peer review: Reviewer response for version 1

**DOI:** 10.21956/wellcomeopenres.17755.r41598

**Published:** 2020-12-15

**Authors:** Gabriele Anton

**Affiliations:** 1 Institute of Epidemiology, Helmholtz Zentrum München, Neuherberg, Germany; 2 German Center for Infection Research (DZIF), partner site, Munich, Germany

With this article the authors present an important contribution to the field of pre-analytical and analytical sample quality. Especially the dilution problem is an issue that presumably occurs in many studies and biobanks but due to smaller sample sizes and/or less rigorous quality controls may stay undetected in many of them. Therefore I would like to thank the authors for this important contribution to overall study quality.

 The approach of UK Biobank to “convert the information contained in the biological samples, which are limited and depletable, into data that can be readily used by researchers worldwide from both academia and industry” is as well highly admirable and will hopefully be kept for the future. By measuring the whole cohort including repeat visit samples at once a number of quality problems like changes in assays, machines or as well differing sample quality due to different storage times can be omitted. These are topics where other cohort studies continuously struggle with and need to invest considerable resources for.

 A few comments/thoughts on the article:

- Why did you choose to measure individual biochemistry markers instead of (MS) metabolomics and proteomics panels?
- Regarding fasting state: your statements on the reason of omitting fasting samples (page 4) is not entirely clear to me; did sampling take place at the same time of the day for all participants and therefore observed time differences to last meal were small in the pilot phase? Did you record the time of last meal for all participants in the main phase?
- The decision to use a central laboratory for sample processing has been described and discussed, but other cohorts worldwide use the decentralized approach and there are articles pointing to changes in samples during transport and storage prior to processing (e.g. references ^1^^-^^3^) – I think it would be good to mention this and cite some of these articles here (page 4).
- From Fig. 1 it seems that the dilution problem from aliquot 1 to 3 always occurs but is stronger in certain time periods (and was detected because of these certain time periods); it would be good to state this more clearly in the text (and abstract) as it is an important information for all laboratories/biobanks working with pipetting robots.
- Stage 2 of the analysis: you start the analysis with 24 (26) assays, but then calculate the estimated sample dilution factor with 17 assays; would be nice to shortly state here, which ones were taken out.
- On page 10 you state: “Assays with a naturally wide biological range are typically far less materially affected from an epidemiological perspective by a given dilution, as small dilution errors are small compared with the biological variation across the population”. This is of course true from a mathematical point of view, but still sounds confusing to me because what is affected by the dilution is the sample and therefore in principle all assays are affected the same and systematically measure false values in addition to biological variation; So maybe complete samples with a certain dilution threshold should be excluded rather than individual assay results?
- To correct for dilution effects it could help to use intra-sample ratios of assay results rather than correct absolute values, at least for assays with similar water-solubility; did you try such approaches?
- Do you have contact to the manufacturers of pipetting robots and/or are you aware of measures to cope with the dilution problem? One could think about including daily dilution controls by aliquoting colored solutions or something the like as additional routine control.

Is the work clearly and accurately presented and does it cite the current literature?

Yes

If applicable, is the statistical analysis and its interpretation appropriate?

Yes

Are all the source data underlying the results available to ensure full reproducibility?

Yes

Is the study design appropriate and is the work technically sound?

Yes

Are the conclusions drawn adequately supported by the results?

Yes

Are sufficient details of methods and analysis provided to allow replication by others?

Yes

Reviewer Expertise:

Biobanking, population cohorts, sample quality.

I confirm that I have read this submission and believe that I have an appropriate level of expertise to confirm that it is of an acceptable scientific standard.

## References

[1] : Strong impact on plasma protein profiles by precentrifugation delay but not by repeated freeze-thaw cycles, as analyzed using multiplex proximity extension assays. *Clinical Chemistry and Laboratory Medicine (CCLM)*.2018;56(4) : 10.1515/cclm-2017-0648 582-594 10.1515/cclm-2017-0648 29040064

[2] : LacaScore: a novel plasma sample quality control tool based on ascorbic acid and lactic acid levels. *Metabolomics*.2016;12: 10.1007/s11306-016-1038-1 96 10.1007/s11306-016-1038-1 27199628PMC4848344

[3] : Differences in metabolite profiles caused by pre-analytical blood processing procedures. *J Biosci Bioeng*.2018;125(5) : 10.1016/j.jbiosc.2017.11.011 613-618 10.1016/j.jbiosc.2017.11.011 29258730

# Response

**Published:** 2020-12-17

**Authors:** Naomi Allen

**Affiliations:** University of Oxford, UK

1. Why did you choose to measure individual biochemistry markers instead of (MS) metabolomics and proteomics panels?

 Response: At the time of the biochemistry study conception (2012), the metabolomic and proteomic assays were not considered mature enough and instead we focused on providing data on key biochemistry markers that would be of high value to a wide range of researchers. UK Biobank is currently undertaking proteomic assays on a subset of the cohort and is keen to expand this to other –omic assays.

 2. Regarding fasting state: your statements on the reason of omitting fasting samples (page 4) is not entirely clear to me; did sampling take place at the same time of the day for all participants and therefore observed time differences to last meal were small in the pilot phase? Did you record the time of last meal for all participants in the main phase?

 Response: Blood collection occurred throughout the day depending on the time of the participant assessment visit, as now outlined in the revised paper. Time since last meal (fasting time) was collected for all 500,000 participants.

 3. The decision to use a central laboratory for sample processing has been described and discussed, but other cohorts worldwide use the decentralized approach and there are articles pointing to changes in samples during transport and storage prior to processing (e.g. references ^1^ ^-^ ^3^) – I think it would be good to mention this and cite some of these articles here (page 4).

 Response: Having a centralised lab for sample processing is far more cost-effective and efficient than having local processing and storage capacity at multiple assessment sites. We performed pilot studies that showed that a very wide range of assays could be performed in samples maintained at 4°C for up to 36 hours prior to processing and storage, as detailed in the paper. As such, the samples were minimally processed at the assessment centres, with most of the processing conducted at the central laboratory using more efficient automated systems. As we recognised the importance of limiting the delay as much as possible, all samples were transported overnight to the central lab and aliquoted and stored within 24 hours. Further, our standardised sample handling protocol ensured that all samples were treated in the same manner and thus will be similarly affected by any pre-analytical sample processing effects, should they exist. We have expanded upon this in the revised paper.

 4. From Fig. 1 it seems that the dilution problem from aliquot 1 to 3 always occurs but is stronger in certain time periods (and was detected because of these certain time periods); it would be good to state this more clearly in the text (and abstract) as it is an important information for all laboratories/biobanks working with pipetting robots.

 Response: This information is included in the Abstract, the Methods and the Results.

 5. Stage 2 of the analysis: you start the analysis with 24 (26) assays, but then calculate the estimated sample dilution factor with 17 assays; would be nice to shortly state here, which ones were taken out.

 Response: Those assays performed in urine (microalbumin, creatinine, postassium, sodium) and red blood cells (HbA1c), plus serum assays with a high biological coefficient of variation (lipoprotein (a), gamma glutamyltransferase, C-reactive protein, rheumatoid factor) plus vitamin D, where seasonal variation masked any dilution effects related to time of sample acquisition, were excluded from the dilution estimation -  leaving 24 assays included. These details are included in Stage 2 of the Methods.

 6. On page 10 you state: “Assays with a naturally wide biological range are typically far less materially affected from an epidemiological perspective by a given dilution, as small dilution errors are small compared with the biological variation across the population”. This is of course true from a mathematical point of view, but still sounds confusing to me because what is affected by the dilution is the sample and therefore in principle all assays are affected the same and systematically measure false values in addition to biological variation; So maybe complete samples with a certain dilution threshold should be excluded rather than individual assay results?

 Response: While it is the sample that is diluted, the impact of this dilution on any subsequent analysis will differ by assay, depending on its natural biological variation. As such, we have recommended that researchers exclude results from those assays that are likely to be highly affected by such dilution, rather than exclude the sample in its entirety. Researchers can, of course, choose to exclude samples above a certain dilution threshold, should they wish to do so.

 7. To correct for dilution effects it could help to use intra-sample ratios of assay results rather than correct absolute values, at least for assays with similar water-solubility; did you try such approaches?

 Response: We did not try any additional approaches beyond those described. Analyses were based on log transformed assay values and so did model proportional effects. As noted, this was a ‘one-size fits all’ approach as a first pass to draw attention to issues before releasing the data and making it available for others to try further approaches

 8. Do you have contact to the manufacturers of pipetting robots and/or are you aware of measures to cope with the dilution problem? One could think about including daily dilution controls by aliquoting colored solutions or something the like as additional routine control.

 Response: This is, of course, possible, but we identified the problem some years after the aliquoting was performed and so couldn’t introduce this measure ourselves. Future studies should consider methods to check for inadvertent dilution due to faulty seals on the equipment.

# Peer review: Reviewer response for version 1

**DOI:** 10.21956/wellcomeopenres.17755.r41121

**Published:** 2020-11-12

**Authors:** Christina Ellervik

**Affiliations:** 1 Faculty of Health and Medical Sciences, University of Copenhagen, Copenhagen, Denmark; 2 Harvard Medical School, Boston, MA, USA

The authors undertake an important task to evaluate the effect of important pre-analytical and analytical and post-analytical factors on the biochemistry variables measured in UK Biobank.

 Although, the focus is UK Biobank, the importance of this work extends to other biobanks and cohorts as well. And given that thousands of studies are approved in UK Biobank and many of them rely on biochemistry variables as exposure, outcomes, or co-variates, identifying “hidden” factors for values of these variables are really important.

 Most investigators are unconsciously ignorant of these factors being important. Therefore, clinical chemistry knowledge, as presented by these authors, is important and pertinent to understand systematic and random variation in the biochemistry variables being measured.

 The authors investigated dilution, regression dilution bias, managing values below the detection limit, and correcting for laboratory drift in assay results.

 What I would like to know more about, which I think is needed to understand the details better in this paper, are:

1. What was the timeline between collection, processing, analyzing, and reporting results (a visual would help)?
2. What time of the day were samples collected?
3. How long time did it take to measure these variables if they were measured on biobanked samples (from which year to year)?
4. Were samples frozen and then biochemistry measured on biobanked samples or was biochemistry measured on fresh samples?
5. You include HbA1c which was measured on red cells, thus, I believe HbA1c must have been measured on fresh samples.
6. Whenever you write about a biomarker in text or tables or figures, please denote “u-“, “p-“, “s-“ , or “WB-“ (for urine, plasma, serum, whole blood) in front of the biomarker. As far as I understand from a previous paper from UK Biobank, glucose was measured on plasma, but other blood-biochemistry biomarkers were measured on serum. WB was for red and white cells and HbA1c (Jackson, 2008) ^1^.
7. Although you refer to the companion paper, I think at least name and generation of instruments for the measurements should be included in this paper (in text or as table).
8. Finally, a visual for the design of this paper (with aliqouts etc.) would be helpful to get an overview of this paper

Is the work clearly and accurately presented and does it cite the current literature?

Yes

If applicable, is the statistical analysis and its interpretation appropriate?

Yes

Are all the source data underlying the results available to ensure full reproducibility?

Yes

Is the study design appropriate and is the work technically sound?

Yes

Are the conclusions drawn adequately supported by the results?

Yes

Are sufficient details of methods and analysis provided to allow replication by others?

Yes

Reviewer Expertise:

Clinical Biochemistry, genetics, population studies, hematology, epidemiology.

I confirm that I have read this submission and believe that I have an appropriate level of expertise to confirm that it is of an acceptable scientific standard.

## References

[1] : UK Biobank Pilot Study: stability of haematological and clinical chemistry analytes. *Int J Epidemiol*.2008;37 Suppl 1: 10.1093/ije/dym280 i16-22 10.1093/ije/dym280 18381388

# Response

**Published:** 2020-12-17

**Authors:** Naomi Allen

**Affiliations:** University of Oxford, UK

1. What was the timeline between collection, processing, analyzing, and reporting results (a visual would help)?

 Response: The biological samples were collected at recruitment (2006-2010) for all 500,000 participants, and at a repeat assessment (2012-2013) for a subset of 20,000 participants. All samples were processed (i.e. aliquoted and stored in a -80°C freezer) within 24 hours of blood collection. Measurement of all 34 assays was phased; the urine samples were assayed between Aug 2014 and Feb 2016, red blood cells (HbA1c) between Oct 2014 and March 2016, and the serum samples between Nov 2015 and Oct 2017. Investigation of the pre-analytical and analytical sources of error was then examined and the data released May 2019. This has been made clearer in the revised article.

 2. What time of the day were samples collected?

 Response: The samples were collected at various times throughout the day, depending on the time that participants attended an assessment centre. This has been added to the revised article.

 3. How long time did it take to measure these variables if they were measured on biobanked samples (from which year to year)?

 Response: See response to Q1.

 4. Were samples frozen and then biochemistry measured on biobanked samples or was biochemistry measured on fresh samples?

 Response: See response to Q1. All of the samples were stored at -80 C until required for assay (as stated in the Methods).

 5. You include HbA1c which was measured on red cells, thus, I believe HbA1c must have been measured on fresh samples.

 Response: HbA1c was measured from packed red blood cells, stored at -80 C until the date of assay.

 6. Whenever you write about a biomarker in text or tables or figures, please denote “u-“, “p-“, “s-“ , or “WB-“ (for urine, plasma, serum, whole blood) in front of the biomarker. As far as I understand from a previous paper from UK Biobank, glucose was measured on plasma, but other blood-biochemistry biomarkers were measured on serum. WB was for red and white cells and HbA1c (Jackson, 2008).

 Response: The haematological markers (e.g., red and white blood cell count) were measured in fresh blood samples at the time of sample collection and are not included in this report (as the dilution issue occurred during processing and aliquoting of the sample prior to long-term storage at -80 C). We’ve now denoted the sample time for each biomarker in revised table 2. Plasma samples were not used for any of these biochemistry assays.  

 7. Although you refer to the companion paper, I think at least name and generation of instruments for the measurements should be included in this paper (in text or as table).

 Response: We’ve included in the revised report an additional table (Table 2) detailing the instruments used to measure each of the biomarkers.

 8. Finally, a visual for the design of this paper (with aliqouts etc.) would be helpful to get an overview of this paper.

 Response: We have provided further detail in the introduction regarding the timelines from data collection through to data analysis and release.
